# The influence of older adults’ digital health literacy on health information protection behavior: a moderated mediation model

**DOI:** 10.3389/fpubh.2026.1754211

**Published:** 2026-03-23

**Authors:** XuYan Liu, RenLong Liang, Jijun Wu, Yin Yuan, YiWei Li, Rui Jian, Heng Ding, Tingting Cai, Yingxin Jia

**Affiliations:** 1 Department of Nursing, Deyang People’s Hospital, Deyang, Sichuan, China; 2 Department of Neurology, Deyang Hospital Affiliated Hospital of Chengdu University of Traditional Chinese Medicine, Deyang, Sichuan, China; 3 Department of Gynecology, Deyang People’s Hospital, Deyang, Sichuan, China; 4 Department of Medical-Nursing Integration, Deyang Hospital Affiliated Hospital of Chengdu University of Traditional Chinese Medicine, Deyang, Sichuan, China

**Keywords:** digital health literacy, family support, health information protection behavior, internet privacy concerns, moderated mediation, older adults

## Abstract

**Objective:**

This study aimed to explore the influencing mechanism of digital health literacy on health information protection behavior among older adults, clarify the mediating role of Internet privacy concerns and the moderating role of family support.

**Methods:**

Convenience sampling was used to select older adults aged 60 years and above with experience in accessing digital health information as respondents, and data were collected through questionnaires. The Digital Health Literacy Scale, Internet Privacy Concerns Scale, Health Information Protection Behavior Scale, and Family Support Scale were used for measurement. SPSS 27.0 and the Process Macro (Models 4 and 14) were employed to conduct descriptive statistics, correlation analysis, and moderated mediating effect tests.

**Results:**

Scores of digital health literacy, health information protection behavior, Internet privacy concerns, and family support among older adults all reached moderate to above-moderate levels. All variables showed a significant positive correlation (all *p* < 0.01). The tests of mediating effect confirmed that Internet privacy concerns played a partial mediating role between digital health literacy and health information protection behavior, accounting for 38.42% of the total effect. Tests of moderating effect showed that family support positively moderated the relationship between Internet privacy concerns and health information protection behavior. Moreover, the mediating effect of Internet privacy concerns gradually strengthened as the level of family support increased.

**Conclusion:**

This study confirms that older adults’ digital health literacy not only directly affects their health information protection behavior, but also exerts an indirect impact through Internet privacy concerns. Meanwhile, family support significantly strengthens the positive effect of Internet privacy concerns on this behavior. The “ability-cognition-behavior” multidimensional driving mechanism identified in this study provides empirical evidence for improving the digital health information protection capacity of older adults. Based on this, we can targetedly promote the integration of digital health literacy and privacy protection education, and build a linked support system of family guidance and community services. This helps older adults translate privacy concerns into practical health information protection actions.

## 1 Introduction

The latest data from the National Bureau of Statistics show that by the end of 2024, the national older population aged 60 and above reached 310.31 million, accounting for 22.0% of the national total population. With the accelerated aging of the population and the popularization of digital health technologies, the demand of older adults for accessing online health information has been steadily increasing. However, risks such as health information leakage and misuse have made their health information protection behavior a focus of attention in the field of digital health (1, 2). Fraud cases caused by the leakage of older adults’ health information have shown a significant upward trend, becoming a key issue restricting the protection of older adults’ digital health rights and interests (3). Existing studies have confirmed that digital health literacy exerts a significant positive impact on individuals’ health information protection behavior (4). As a cognitive mediating variable, Internet privacy concerns have been partially verified to play a mediating role between information literacy and information protection behavior (5). Meanwhile, family support, as a key dimension of social support, has also been proven to moderate the effect of digital literacy on health-related behaviors of older adults (6). However, most existing studies focus on direct relationships between individual variables and fail to systematically reveal the complex pathways through which digital health literacy influences health information protection behavior, resulting in a lack of precision in intervention measures. By integrating core variables to construct a multi-path model, this study not only enriches the theoretical foundation but also provides a targeted empirical basis for formulating differentiated intervention strategies for older adults’ health information protection, thus holding significant practical application value.

### 1.1 The relationship between older adults’ digital health literacy and health information protection behavior

Digital health literacy refers to individuals’ the core ability to access, understand, evaluate, and apply health information and services in the digital environment. It includes three dimensions: Ability to Apply Online Health Information and Services, Critical Evaluation Ability, and Health Decision-Making Ability, and serves as the fundamental capacity for realizing digital health rights and interests. Health information protection behavior refers to a series of proactive actions taken by individuals to prevent risks such as health information leakage and misuse, and is a concrete manifestation of health rights protection in the digital health environment.

The Theory of Planned Behavior (TPB) states that an individual’s cognitive level influences specific behavioral performance by affecting behavioral attitudes and subjective norms (7). For older adults, digital health literacy directly determines their ability to identify and respond to health information risks (8). Empirical studies have shown that the higher the digital health literacy score of respondents, the more cautious they are when facing online health information, and the more active their health information screening and protection behaviors become (9). Yang et al. (10) found that health information literacy not only helps older adults identify health rumors to avoid their spread but also prevents them from making incorrect health decisions due to false information. A scoping review including 35 studies revealed that improving participants’ digital information literacy can enhance their ability to distinguish health information, awareness of privacy protection, and risk perception of inappropriate sharing—confirming that enhancing digital health literacy contributes to better health information protection behaviors (11).

Although not all subjects in the above studies are older adults, based on the aforementioned theoretical and empirical findings, we reasonably infer that digital health literacy still exerts a positive predictive effect on health information protection behavior among older adults. Thus, the following hypothesis is proposed:

*H1*: Older adults’ digital health literacy is significantly positively correlated with their health information protection behavior.

### 1.2 The mediating role of internet privacy concerns

Internet privacy concerns refer to older adults’ risk perception and subjective worries regarding the collection, use, sharing, and other processes of their personal information in the online environment. It specifically includes three dimensions: Privacy Control Perception, Privacy Awareness Perception, and Privacy Collection Perception, and serves as a key psychological variable linking cognition and behavior.

**Figure 1:**
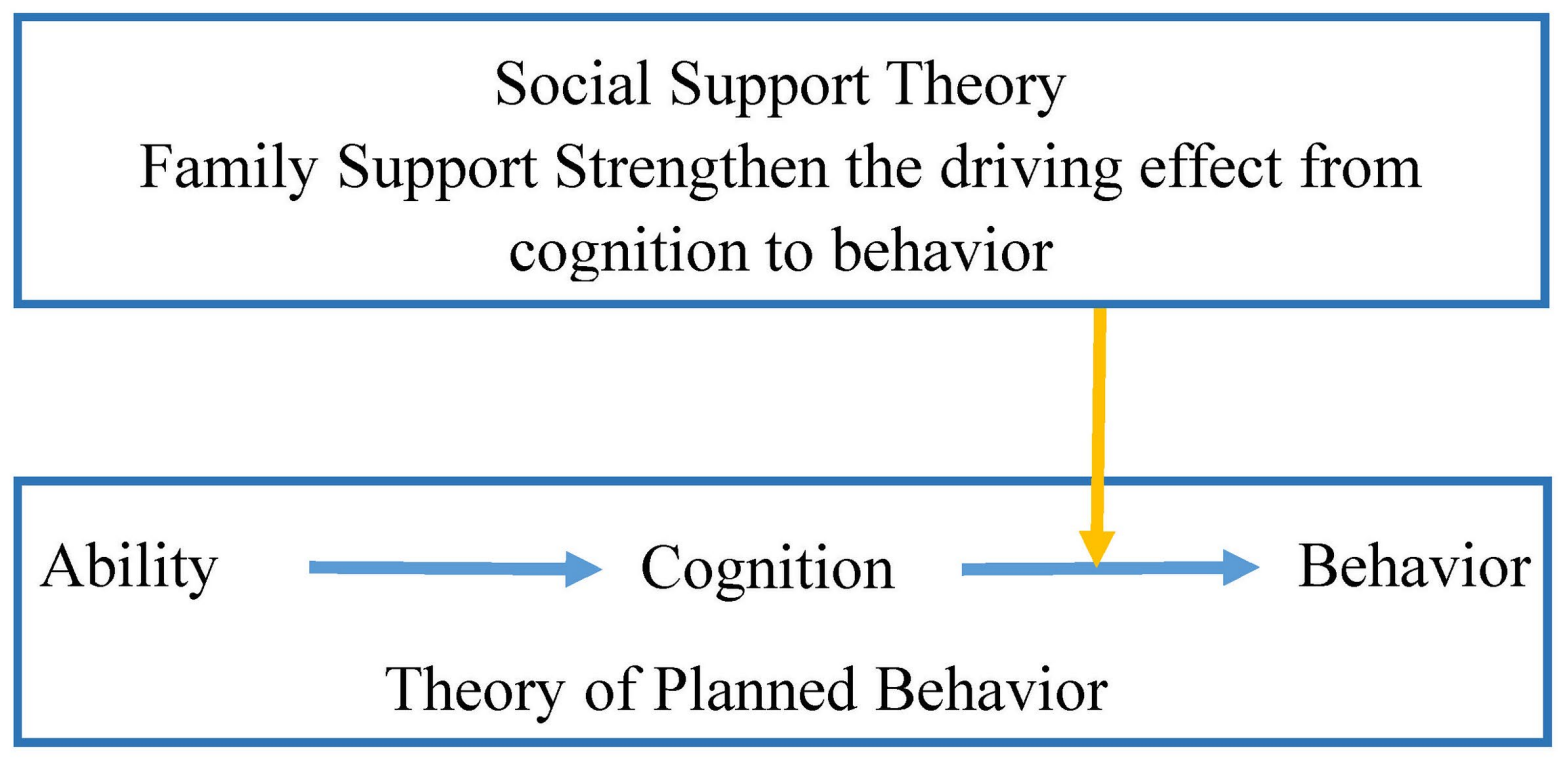
Theoretical framework diagram.

The cognitive pathway of the Theory of Planned Behavior (TPB) further emphasizes that an individual’s cognitive ability ultimately influences specific behaviors by shaping behavioral attitudes and risk perception, forming a theoretical logical chain from ability to cognition to behavior (see Figure 1) (12). Based on this theory, as older adults’ subjective cognitive evaluation of online privacy risks, Internet privacy concerns may serve as a key bridge connecting digital health literacy and health information protection behavior. On the one hand, older adults with high digital health literacy are more likely to perceive the risk of online health information leakage, thereby enhancing their level of privacy concerns (13); on the other hand, older adults with higher levels of Internet privacy concerns are more inclined to adopt protective behaviors such as information encryption and cautious sharing (14). In terms of empirical research, a study by Erdat et al. on 210 nursing interns showed that digital health literacy is significantly positively correlated with Internet privacy concerns (15). A qualitative study by Grande et al. (16), which included 45 American consumers, revealed that respondents generally did not know that consumers’ digital data might reveal their health status—indicating insufficient Internet privacy concerns and a greater lack of health information protection behaviors among these respondents. However, all respondents expressed a desire for their health information to be protected.

In summary, existing studies have initially identified the mediating effect of privacy concerns between information literacy and information protection behavior in the general population, but specialized verification for the older adult group remains scarce. Based on this, the following hypotheses are proposed:

*H2*: Older adults’ digital health literacy is positively correlated with their Internet privacy concerns.

*H3*: Older adults’ Internet privacy concerns are positively correlated with their health information protection behavior.

*H4*: Older adults’ Internet privacy concerns play a mediating role between their digital health literacy and health information protection behavior.

### 1.3 The moderating role of family support

Social Support Theory holds that social support can influence the behavioral decision-making process by moderating the strength of the relationship between an individual’s cognition and behavior (17). As the core source of social support for older adults (18), family support can provide them with information guidance, emotional encouragement, and behavioral assistance, thereby strengthening the driving effect of cognitive factors on behavior (see Figure 1) (19). In short, family support can actively transform concerns about Internet privacy into proactive protective behaviors. A survey study involving 214 older adults showed that older adults’ perception of involvement in online privacy risks significantly affects their privacy protection behaviors (20). It also pointed out that family members’ technical help and emotional encouragement are key factors to promote the transformation of risk perception into actual behavior. Older adults with higher levels of family support are more likely to transform their cognition of online privacy risks into practical information protection behaviors (21). In contrast, those with insufficient family support—even if they have a certain level of privacy concerns—may struggle to implement protective behaviors due to insufficient operational ability or lack of motivation (22, 23). This implies that family support may moderate the relationship between Internet privacy concerns and health information protection behavior, and further influence the strength of the mediating effect. Based on this, the following hypothesis is proposed:

*H5*: Family support positively moderates the effect of Internet privacy concerns on health information protection behavior in the mediating model.

### 1.4 Construction of the hypothetical model and research significance

**Figure 2:**
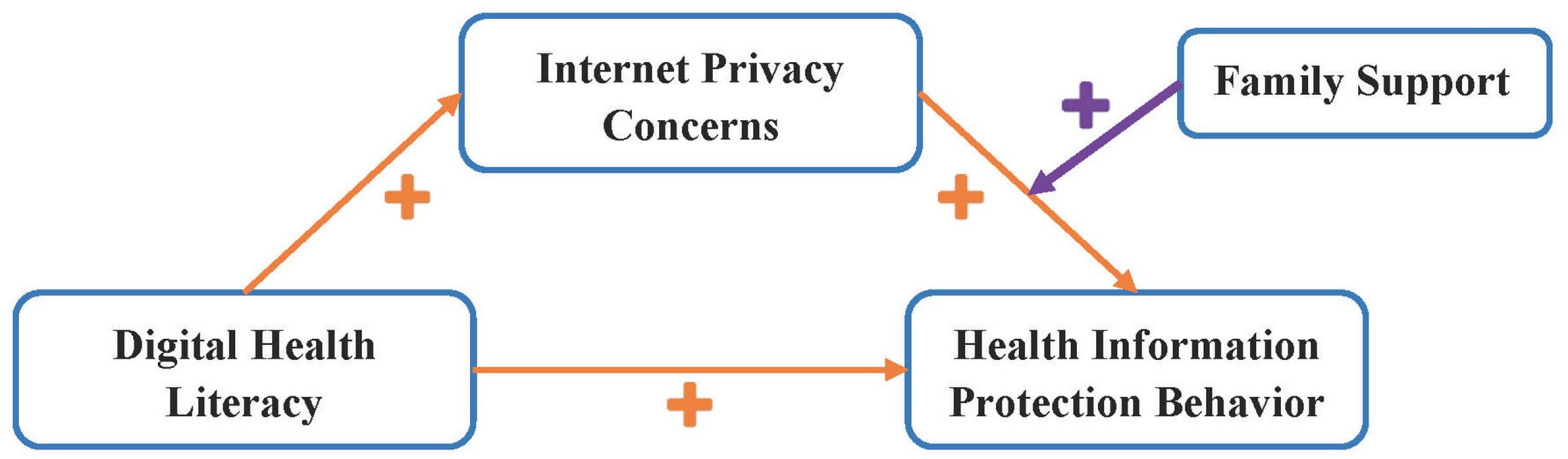
Hypothetical model diagram.

Based on the TPB (7) and Social Support Theory (17), digital health literacy may influence older adults’ health information protection behavior through Internet privacy concerns, while family support may moderate the strength of this mediating pathway. However, existing studies have not incorporated these four variables into a unified moderated mediation framework, making it difficult to systematically reveal the complex influence mechanism of older adults’ health information protection behavior. Therefore, this study constructs a moderated mediation model with Internet privacy concerns as the mediating variable and family support as the moderating variable (see Figure 2). It aims to provide a theoretical supplement and empirical evidence for understanding the formation mechanism of older adults’ health information protection behavior.

## 2 Materials and methods

### 2.1 Study design and sample size calculation

This study adopted a cross-sectional design and adhered to the STROBE guidelines. From March to May 2025, older adults were randomly recruited from 5 communities for the survey using the convenience sampling method. The sample size was calculated using G*Power 3.1 software (24). The significance level was set at 0.05, statistical power at 95%, a medium effect size (f^2^ = 0.15) was used, and there were a total of 14 predictor variables in this study. Based on the above parameters, the minimum required effective sample size was calculated to be 194. Considering the large differences in older adults’ educational levels and the potential low completeness of questionnaire responses, an additional 15–20% of samples were reserved compared with the general population to avoid insufficient effective samples. Meanwhile, to ensure the credibility of the results, this study included as many samples as possible under available conditions, with a final planned sample size of 450 older adults.

### 2.2 Study participants

The inclusion criteria for this study are as follows: Aged 60 years or older; Having experience in accessing health information via digital devices (such as smartphones, computers, and tablets) in the past 6 months; Having fixed family interaction partners; No severe cognitive impairment; Able to complete the questionnaire independently or with the assistance of researchers.

The exclusion criteria for this study are as follows: Individuals unable to provide accurate age information; Diagnosed with cognitive impairment or severe hearing/speech impairments that prevent understanding of the questionnaire content; Individuals unable to communicate normally; Missing response rate exceeding 10% during questionnaire completion.

### 2.3 Ethical considerations

This study complied with The Declaration of Helsinki and was approved by the Ethics Committee of Deyang Hospital Affiliated to Chengdu University of Traditional Chinese Medicine. Before the survey began, the study’s purpose, significance, and inclusion criteria were explained to the participants. The survey was conducted only after obtaining the participants’ consent. To protect the participants’ privacy, this study did not collect their names, home addresses, or contact information.

### 2.4 Research tools

#### 2.4.1 General information survey form

This questionnaire was developed based on previous literature reviews. It includes the following variables: gender, age, educational level, whether living with children, monthly disposable expenses, daily usage duration of intelligent devices, whether suffering from chronic diseases, channels for obtaining online health information, and self-rated health status.

#### 2.4.2 Digital health literacy

The simplified Chinese version of the eHealth Literacy Scale (SC-eHEALS) was used for the survey. This scale was originally developed by Norman (25) and later underwent simplified Chinese translation and cultural adaptation by Guo et al. (26). It consists of 3 dimensions and 8 items, including the Ability to Apply Online Health Information and Services (5 items), Critical Evaluation Ability (2 items), and Health Decision-Making Ability (1 item). A 5-point Likert scale was adopted, with scores ranging from 1 (“strongly disagree”) to 5 (“strongly agree”). The total score of the scale ranges from 8 to 40, with higher scores indicating higher levels of digital health literacy. Further psychometric evaluation of the SC-eHEALS in the Chinese population showed that its Cronbach’s *α* coefficient was 0.96, McDonald’s *ω* was 0.92, and Composite Reliability was 0.96 (27), confirming that the eHEALS has good reliability and validity among the Chinese population. The Cronbach’s α coefficient of this scale in the current study was 0.812.

#### 2.4.3 Health information protection behavior

Based on the TPB, the Health Information Protection Behavior Scale for Older Adults was constructed by integrating the measurement method of Internet privacy protection behavior by Smith et al. (28) and the Privacy Behavior Scale developed by Li (29), with adjustments made to fit the context of older adults. This scale includes three dimensions and 12 items in total: Health Data Authorization and Management (4 items), Sensitive Information Sharing Control (4 items), and Seeking Family and Social Support (4 items). A 5-point Likert scale was used, with scores from 1 (“strongly disagree”) to 5 (“strongly agree”). The total score ranges from 12 to 60, with higher scores indicating better health information protection behavior.

The KMO measure of sampling adequacy for this scale was 0.862, and Bartlett’s test of sphericity yielded an approximate chi-square value of 1347.911 (df = 66, *p* < 0.001), indicating suitability for factor analysis. Exploratory factor analysis showed that the factor loadings of all items ranged from 0.474 to 0.651, with a cumulative variance explanation rate of 57.73%. The Cronbach’s *α* coefficients of the dimensions ranged from 0.714 to 0.746, and the Cronbach’s α coefficient of the total scale was 0.838, meeting psychometric standards.

#### 2.4.4 Internet users’ information privacy concerns

The Internet Users’ Information Privacy Concerns (IUIPC) scale was developed by Malhotra et al. (30) in 2004 and later optimized into an 8-item version (IUIPC-8). It includes 3 core dimensions: Control (2 items), Awareness (2 items), and Collection (4 items) (31). Compared with the IUIPC-10, the optimized IUIPC-8 has higher reliability and validity and is suitable for research scenarios involving disease-related privacy of older adults. A 7-point Likert scale was used for this scale, with scores from 1 (“strongly disagree”) to 7 (“strongly agree”). The total score ranges from 8 to 56, with higher scores indicating higher levels of privacy concerns. The Cronbach’s *α* coefficient of this scale in the current study was 0.848.

#### 2.4.5 Family support

Family support was measured using the family support dimension of the Multidimensional Scale of Perceived Social Support (MSPSS). The MSPSS was developed by Zimet et al. (32) and later refined by Reeve et al. (33). It includes 3 dimensions (family support, friend support, and support from other significant others) with 4 items per dimension, totaling 12 items. A 7-point Likert scale was adopted, with scores from 1 (“strongly disagree”) to 7 (“strongly agree”). Higher scores indicate higher levels of perceived support, and the Cronbach’s *α* coefficient of the full scale is 0.898. Only the family support dimension was used in this study, with a Cronbach’s α coefficient of 0.870.

### 2.5 Data collection and quality control methods

Contact was made with community administrators to explain the purpose and significance of the study, and their approval and assistance were obtained. Researchers distributed paper questionnaires in crowded public areas of the communities. The questionnaire included detailed descriptions of the study’s purpose, significance, and inclusion/exclusion criteria, and respondents were informed of their right to voluntary participation. Meanwhile, respondents were told that their relevant information would be strictly confidential, used only for research purposes, and that sensitive information (such as home addresses, contact numbers, and personal identification information) would not be collected. Researchers provided assistance to respondents during questionnaire completion to ensure accurate responses. Two researchers carefully reviewed and entered the collected questionnaire data, excluding questionnaires with incomplete responses or patterned responses. During the questionnaire design and data collection phases, measures such as anonymous completion, randomized item order, and double data entry with verification were adopted to ensure the reliability of the research results.

### 2.6 Statistical analyses

Statistical analyses were performed using SPSS 27.0. Respondents’ general information was described using frequencies and percentages. Since the sample size met the requirements of the central limit theorem, scale scores were described using mean ± standard deviation. Pearson correlation analysis was used to test the correlations between variables. Harman’s single-factor analysis of variance was conducted to detect common method bias. Hayes’ Process Macro v4.2 (Model 4) was used to verify the mediating effect of Internet privacy concerns between digital health literacy and health information protection behavior. Model 14 in Process v4.2 was used to verify the moderating effect of family support in the mediation model. Simple slope analysis was used to analyze the moderating effect and generate a simple slope plot. The bootstrapping method (*n* = 5,000) was used to test the validity of the mediating effect and moderated mediating effect. If the 95% confidence interval (95% CI) did not include zero, the effect was considered significant. The significance level was set at *α* = 0.05 (34).

## 3 Results

### 3.1 Common method bias test

Harman’s single-factor analysis of variance was used to test for common method bias. The results showed that the eigenvalues of all 7 factors were greater than 1, and the variance explained by the first factor was 27.60%—which did not reach the critical value of 40%. This indicates that there is no significant common method bias in this study.

### 3.2 General information of the respondents

In this study, a total of 448 questionnaires were collected. Among them, 12 were excluded for failing to meet the inclusion/exclusion criteria, 20 for incomplete responses, and 14 for obviously unreasonable responses. Finally, 402 valid questionnaires were included for analysis, with an effective recovery rate of 89.73%. Detailed demographic information of the respondents is shown in Table 1.

**Table 1:** Detailed information on control variables (*n* = 402).

Variables	Category	Number	Percentag (%)
Gender	Male	182	45.3
	Female	220	54.7
Age (years)	60 ~ 69	255	63.4
	70 ~ 79	112	27.9
	≥80	35	8.7
Household registration place	Urban	316	78.6
	Rural	86	21.4
Educational level	Primary school and below	103	25.6
	Junior high school	118	29.4
	Senior high school	117	29.1
	College degree and above	64	15.9
Monthly disposable expenses	≤1,000	62	15.4
	1,001 ~ 3,000	215	53.5
	3,001 ~ 5,000	83	20.6
	>5,000	42	10.4
Daily usage duration of intelligent devices (hours)	≤1	31	7.7
	1 ~ 3	189	47.0
	4 ~ 5	124	30.8
	>5	58	14.4
Whether living with children	Yes	299	74.4
	No	103	25.6
Whether suffering from chronic diseases	Yes	226	56.2
	No	176	43.8
Channels for obtaining online health information	Formal health APP	89	22.1
	Short video platform	110	27.4
	Forwarded by relatives and friends	58	14.4
	AI search	145	36.1
Self-rated health status	Poor	135	33.6
	Fair	206	51.2
	Good	61	15.2

### 3.3 Correlation analysis between variables

Scale scores of variables were described using mean ± standard deviation. Specifically, the score of digital health literacy was 22.56 ± 3.89, health information protection behavior was 35.85 ± 4.42, Internet privacy concerns was 32.06 ± 4.22, and family support was 17.00 ± 3.43. Detailed scores of each variable and its dimensions are presented in Table 2. Spearman correlation analysis was used to examine the correlations between the scales. The results showed that: Digital health literacy was positively correlated with Internet privacy concerns (*r* = 0.575, *p* < 0.01); Digital health literacy was positively correlated with health information protection behavior (*r* = 0.502, *p* < 0.01); Digital health literacy was positively correlated with family support (*r* = 0.218, *p* < 0.01); Internet privacy concerns was positively correlated with health information protection behavior (*r* = 0.514, *p* < 0.01); Internet privacy concerns was positively correlated with family support (*r* = 0.205, *p* < 0.01); Family support was positively correlated with health information protection behavior (*r* = 0.582, *p* < 0.01). Detailed information is shown in Table 3.

**Table 2:** Detailed score information of each variable and its corresponding dimensions (*n* = 402).

Variables/dimensions	Items	Score range	Score situation
Digital health literacy	8	8 ~ 40	22.56 ± 3.89
Ability to apply online health information and services	5	5 ~ 25	14.02 ± 2.64
Critical evaluation ability	2	2 ~ 10	5.56 ± 1.31
Health decision-making ability	1	1 ~ 5	2.99 ± 0.96
Health information protection behavior	12	12 ~ 60	35.85 ± 4.42
Health data authorization and management	4	1 ~ 20	11.86 ± 1.70
Sensitive information sharing control	4	1 ~ 20	11.59 ± 1.81
Seeking family and social support	4	1 ~ 20	12.40 ± 1.97
Internet privacy concerns	8	8 ~ 56	32.06 ± 4.22
Perception of privacy control	2	2 ~ 14	7.46 ± 1.19
Perception of privacy awareness	2	2 ~ 14	8.05 ± 1.39
Perception of privacy collection	4	4 ~ 28	16.55 ± 2.36
Family support	4	4 ~ 28	17.00 ± 3.43

**Table 3:** Correlation analysis between variables.

Variable	Digital health literacy	Health information protection behavior	Internet privacy concerns	Family support
Digital health literacy	1	–	–	–
Health information protection behavior	0.502^**^	1	–	–
Internet privacy concerns	0.575^**^	0.514^**^	1	–
Family support	0.218^**^	0.582^**^	0.205^**^	1

^**^, at the 0.01 level (two-tailed), the correlation is significant.

### 3.4 The mediating effect of internet privacy concerns

To verify the mediating effect of Internet privacy concerns on the relationship between digital health literacy and health information protection behavior, this study used Model 4 in the PROCESS v4.2 Macro to construct a mediating effect model. As shown in Table 4, digital health literacy had a significant direct effect on health information protection behavior (*B* = 0.351, *p* < 0.001), with a 95% confidence interval (CI) of (0.239–0.463). Additionally, the indirect effect mediated by Internet privacy concerns was also significant (*B* = 0.219, *p* < 0.001), with a 95% CI of (0.125–0.332). The mediating effect accounted for 38.42% of the total effect.

**Table 4:** The mediating effect of internet privacy concerns between digital health literacy and health information protection behavior.

Project	Effect value	*SE*	LLCI-ULCI	*p*	Relative effect value (%)
Direct effect	0.351	0.057	0.239 ~ 0.463	0.000	61.58%
Indirect effect	0.219	0.053	0.125 ~ 0.332	0.000	38.42%
Total effect	0.570	0.049	0.474 ~ 0.667	0.000	100.00%

### 3.5 The moderating effect of family support

To verify the moderating effect of family support in the mediation model, hierarchical regression analysis was conducted after data centering, with demographic information included as control variables. Subsequently, Model 14 in the PROCESS v4.2 Macro was used to construct and validate the moderating effect model. Stepwise regression analysis of each variable showed (see Table 5) that: Older adults’ digital health literacy significantly and positively predicted health information protection behavior (*β* = 0.231, *p* < 0.001); Internet privacy concerns significantly and positively predicted health information protection behavior (*β* = 0.279, *p* < 0.001); Family support significantly and positively predicted health information protection behavior (*β* = 0.466, *p* < 0.001); Family support significantly and positively moderated the predictive effect of Internet privacy concerns on health information protection behavior (*β* = 0.096, *p* < 0.01).

**Table 5:** Hierarchical regression analysis testing the moderating effect of family support.

Variable	Model 1			Model 2		
	*β*	*SE*	*P*	*β*	*SE*	*P*
Gender	−0.007	0.304	0.831	−0.013	0.301	0.703
Age	0.068	0.264	0.047	0.065	0.262	0.056
Household registration place	0.121	0.382	<0.001	0.121	0.378	<0.001
Educational level	−0.003	0.182	0.950	−0.012	0.181	0.778
Monthly disposable expenses	0.029	0.219	0.492	0.032	0.217	0.438
Daily usage duration of intelligent devices (hours)	0.029	0.228	0.504	0.031	0.226	0.472
Whether living with children	−0.024	0.477	0.615	−0.030	0.473	0.529
Whether suffering from chronic diseases	0.016	0.306	0.639	0.016	0.303	0.638
Channels for obtaining online health information	0.029	0.180	0.545	0.029	0.179	0.539
Self-rated health status	−0.038	0.320	0.433	−0.040	0.317	0.405
Digital health literacy	0.231	0.049	<0.001	0.236	0.048	<0.001
Internet privacy concerns	0.279	0.044	<0.001	0.278	0.044	<0.001
Family support	0.466	0.045	<0.001	0.483	0.046	<0.001
Internet privacy concerns × family support				0.096	0.009	0.005
*R* ^2^	0.559			0.568		
*∆Rss* ^2^	0.544			0.552		
*F*	137.792^***^			7.795^**^		

^**^P < 0.01, ^***^p < 0.001.

The results indicated that when family support was at the M-SD, M, and M + SD levels, the mediating effect of Internet privacy concerns was significant in all cases, with 95% confidence intervals (95% CI) excluding zero. As the family support received by older adults increased, the mediating role of Internet privacy concerns between digital health literacy and health information protection behavior gradually strengthened, with the total effect value increasing from 0.128 to 0.238 (see Table 6 for details).

**Table 6:** The influence of the moderating variable on the mediating effect value.

Level of family support	Mediating effect size	BootSE	*LLCI*	*ULCI*
Low (−1SD)	0.128	0.053	0.030	0.236
Medium (mean)	0.183	0.048	0.099	0.290
High (+1SD)	0.238	0.055	0.146	0.364

**Figure 3:**
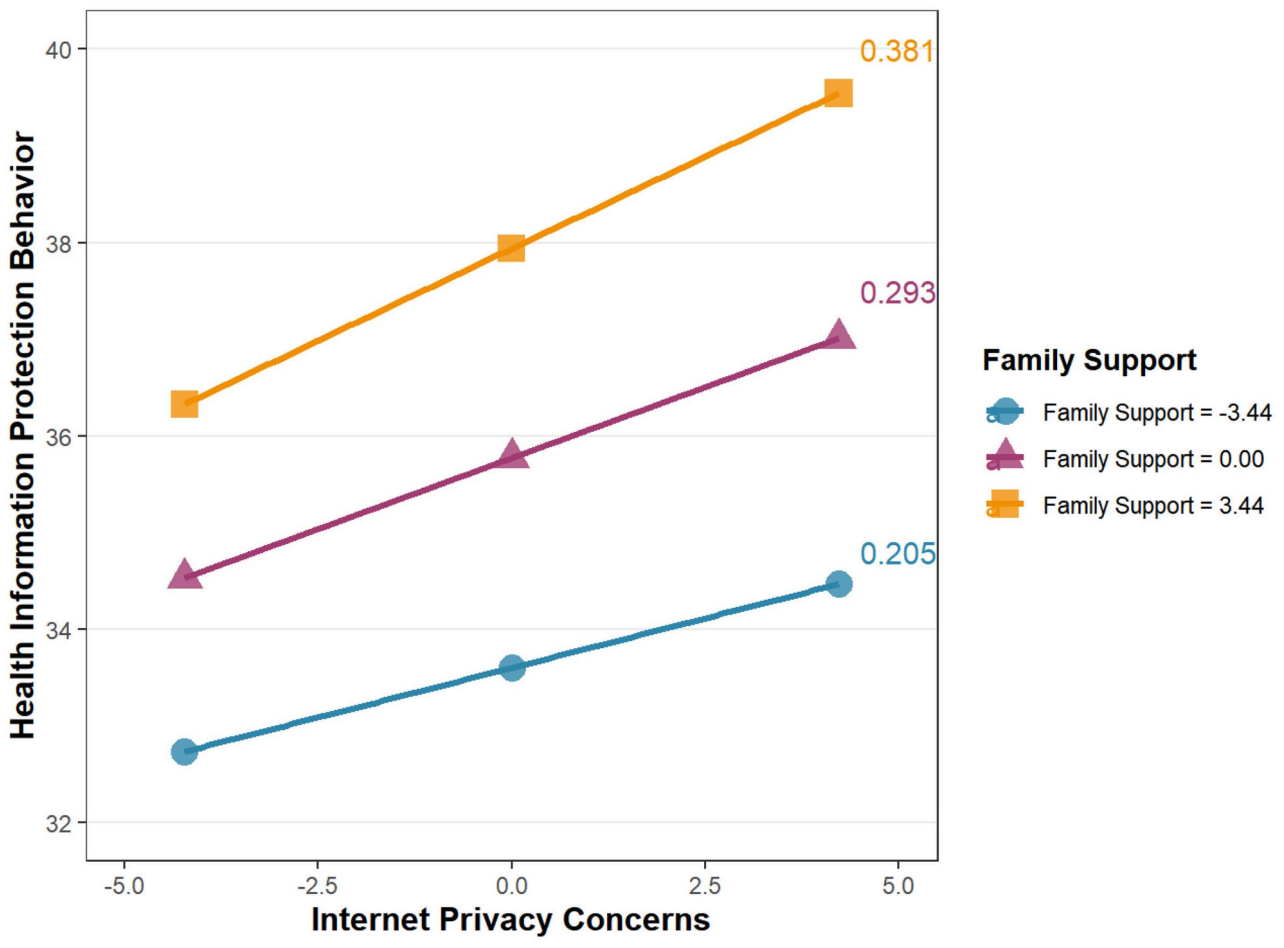
Simple slope plot of the moderating effect.

To further visually observe the moderating effect of family support on the relationship between Internet privacy concerns and health information protection behavior in the mediation model, the simple slope method was used for analysis, and R language was employed to generate a simple slope plot. The results are shown in Figure 3. When family support was at a low level, Internet privacy concerns positively predicted health information protection behavior (*βsimple* = 0.205, *p* < 0.001); When family support was at a moderate level, Internet privacy concerns positively predicted health information protection behavior (*βsimple* = 0.293, *p* < 0.001); When family support was at a high level, Internet privacy concerns still positively predicted health information protection behavior (*βsimple* = 0.381, *p* < 0.001). In other words, as family support increased, the positive predictive effect of Internet privacy concerns on health information protection behavior gradually strengthened. This indicates that family support played a positive moderating role in the impact of Internet privacy concerns on health information protection behavior.

## 4 Discussion

### 4.1 Current status of digital health literacy and related variables among older adults

The results of this study indicated no statistically significant differences in digital health literacy and family support scores between participants with urban and rural household registration, which is obviously inconsistent with reality. On the one hand, the coverage of digital health infrastructure and the penetration of smart devices in rural areas are far lower than those in urban areas. Rural older adults have a more single channel to access digital health information and lack a supportive external environment for cultivating digital health literacy, and this context differs significantly from the digital exposure experience of the urban community-dwelling older adults in this study. On the other hand, the family support model for rural older adults is dominated by traditional offline care. The frequency and depth of intergenerational digital support provided by their children are much lower than those in urban families, suggesting that the facilitating effect of family support on the translation of privacy concerns into protective behaviors may be weaker than the findings of this study. All participants in this study were recruited from urban communities. Although some older adults had rural household registration, they had resided in urban areas long-term, which explains the non-significant score differences. This research limitation reduces the generalizability of the findings. Future research should include a larger sample of individuals who have long resided in rural areas to conduct comparative studies.

In this study, The overall score of older adults’ digital health literacy was at a moderate level (22.56 ± 3.89, full score = 40); The score of health information protection behavior was at an above-moderate level (35.85 ± 4.42, full score = 60); The score of Internet privacy concerns was at an above-moderate level (32.06 ± 4.22, full score = 56); The score of family support was at an above-moderate level (17.00 ± 3.43).

None of the above variable scores were high, and this status reflects the dual dilemmas faced by the current older adult group in the wave of digital health. On the one hand, with the digital transformation of healthcare (e.g., popularization of online registration and consultation, electronic medical records), older adults’ dependence on digital health tools has increased. However, their insufficient digital health literacy has trapped them in the awkward situation of wanting to use these tools well but failing to do so (35). For example, some older adults cannot identify phishing health websites, which not only makes it difficult for them to obtain valid information but also exposes them to the risk of privacy leakage (36). On the other hand, there is passivity in their health information protection behavior: most older adults do not proactively protect their health information (e.g., never proactively modifying APP privacy and permission settings) and rely more on family and social support (37, 38). This is closely related to the older adults digital behavioral inertia of prioritizing practicality over protection, and this problem is more prominent among older adults living alone due to lack of family support (39).

To address this, a multi-dimensional intervention system of “capacity enhancement, risk early warning, and support empowerment” needs to be established: Conduct hierarchical teaching in community digital health classes, and provide practical training on password setting and permission management for older adults with low literacy; Simultaneously push short videos of typical cases of health information protection on social platforms for older adults; For older adults living alone or in older care institutions, community volunteers or older care service staff should serve as “digital companions” to fill the gap in family support.

### 4.2 The impact of digital health literacy on health information protection behavior

This study verified a significant positive correlation between older adults’ digital health literacy and health information protection behavior (*β* = 0.502, *p* < 0.001), thus supporting Hypothesis 1. This result reflects the core logic of “ability determining behavior” in the Theory of Planned Behavior (7): older adults with high digital health literacy can not only access health information efficiently but also proactively protect their privacy through a complete chain of risk identification, strategy selection, and behavior implementation (8, 40).

From a social reality perspective, this mechanism directly points to the paradox of digital health risks faced by the older adult group: the more they rely on digital channels to obtain health information, the more they need protective capabilities. However, their insufficient literacy leads to a situation where “the more dependent they are, the more vulnerable they become.” For example, some older adults do not understand the meaning of health APP permission requests and blindly grant authorization for location information and medical record data, making them easy targets of online fraud (41).

To resolve this paradox, efforts should start from the root causes: First, conduct training to improve older adults’ digital health literacy, cultivate their health information protection skills, and enhance core competencies such as “identifying loopholes in privacy clauses” and “refusing excessive information collection” (42); Second, health service platforms should fulfill their aging-friendly privacy protection responsibilities, add privacy reminders for the older adult mode in product design, disable non-essential information collection permissions by default, and promote the implementation of older-friendly privacy designs on health platforms to lower the operational threshold for older adults (43).

### 4.3 The mediating role of internet privacy concerns: a key bridge for cognitive transformation

Internet privacy concerns exhibited a partial mediating effect between digital health literacy and health information protection behavior (mediating effect value = 0.219, 95% CI [0.125, 0.332]), supporting Hypothesis 4. This mediating effect accounted for 38.42% of the total effect, indicating that digital health literacy not only directly drives protective behavior but also enhances older adults’ cognition of health privacy (e.g., recognizing that health data leakage may lead to targeted fraud) to stimulate their motivation for protection.

This finding addresses the social concern about older adults’ privacy cognition blind spots. Many older adults possess basic digital skills—such as using WeChat for registration and consultation—but due to insufficient awareness of privacy risks, behaviors like directly forwarding health information from doctors to WeChat groups or checking medical records over public WiFi are common occurrences (44). Essentially, this reflects a disconnection between their ability to use digital tools and their ability to recognize risks.

Targeted strategies should focus on the transformation from cognition to behavior: Add a privacy education module to digital health literacy training, and use scenario simulations to help older adults intuitively perceive risks (e.g., simulating classic cases where leakage of disease information or medication records leads to receiving fraudulent health product promotions) (45). Develop a toolkit for transforming privacy concerns into actions—for instance, reminding older adults to verify the authenticity of promotional information they receive or consult their children for confirmation when in doubt. In this way, abstract cognition can be converted into actionable behavioral guidelines. Governments and communities should further incorporate the protection of older adults’ health information into the key priorities of aging-friendly digital policies, formulate the Guidelines for the Protection of Older Adults’ Health Information Privacy, rely on community older care service centers to establish privacy protection service stations, and provide free services such as APP permission checks and password security assessments.

### 4.4 The moderating role of family support: the strengthening effect of social support

Family support significantly and positively moderated the relationship between Internet privacy concerns and health information protection behavior (interaction term *β*: 0.026, *p* < 0.01). Moreover, in the high family support group, the mediating effect of Internet privacy concerns was stronger, and Hypothesis 5 was verified. This indicates that family support serves as an important catalyst for transforming privacy concerns into protective behaviors. Reminders and assistance from children or relatives can effectively compensate for the deficiencies in older adults’ operational abilities (46). For example, helping older adults disable unnecessary permissions of mobile apps can significantly reduce app information push and data collection, thereby preventing the leakage of older adults’ health information and protecting them from fraud (47).

This result highlights the irreplaceability of family digital guardianship in the protection of older adults’ health (48). In contemporary society, the problem of support deficiency caused by the digital generation gap is particularly prominent: some children neglect to provide guidance due to busy work, or believe that older adults should use mobile phones less because they are not proficient in them, which instead exacerbates their information isolation (49).

Constructing a collaborative support network involving families and society is the key to breaking this dilemma. First, implement digital feedback incentive programs, such as communities providing older care service points to children who teach their parents privacy protection skills; Second, strengthen the awareness of digital feedback, regularly assist parents in updating privacy settings and clearing sensitive information, improve their protection capabilities through hands-on teaching combined with case warnings, and convert family support into sustainable protection motivation; Third, establish a “family support combined with community services” linkage mechanism. For older adults with insufficient family support, communities or older care institutions shall provide one-on-one guidance on privacy settings to ensure that high privacy concerns do not degenerate into helpless inaction; Fourth, encourage older adults to actively participate in community digital skills training, develop the usage habit of checking permissions before filling in information, and promptly seek help from children or community volunteers when encountering uncertain health information sharing scenarios, so as to avoid neglecting privacy protection due to reluctance to take trouble.

## 5 Study limitations

Limitation in Sample Representativeness: The sample of this study was primarily derived from older adults residing in urban communities, with a significant underrepresentation of rural older adults. Substantial differences exist between urban and rural older populations in terms of access to digital infrastructure and family support models. This sample characteristic may reduce the generalizability of the research results. Furthermore, all study participants were recruited from Deyang City, Sichuan Province. As a prefecture-level city, Deyang’s level of digital health development, characteristics of older digital literacy, and family support models differ to some extent from those of coastal eastern China and other provinces in the central and western regions. Consequently, the research conclusions may not be directly generalizable to other areas nationwide. Future research should expand the inclusion proportion of rural samples, conduct comparative studies between urban and rural areas, and further broaden the geographical coverage of the study to enhance the generalizability of the findings.

Limitation in Causal Inference: This study adopted a cross-sectional survey design. Although variable relationships were established through theoretical deduction, it could not fully verify the dynamic causal chain between variables. Future studies need to further verify this through longitudinal studies.

Limitation in Variable Measurement Details: On the one hand, the data on health information protection behaviors in this study were all self-reported by the older adults. Influenced by factors such as respondents’ subjective cognition and social desirability bias, the data results may deviate from their actual health information protection actions, failing to fully reflect their real behavioral performance. On the other hand, although the Internet privacy concerns scale used in this study passed the reliability and validity tests, it did not distinguish between privacy concerns regarding health information and non-health information. This may obscure the particularities of older adults’ protection behaviors specific to different types of information. Subsequent studies can optimize the measurement tools to improve the precision of variable measurement.

## 6 Conclusion

Through the verification of a moderated mediation model, this study found that: Older adults’ digital health literacy not only directly and positively affects health information protection behavior but also indirectly influences this behavior through the mediating role of Internet privacy concerns; Furthermore, family support can strengthen the positive effect of Internet privacy concerns on health information protection behavior—specifically, the higher the level of family support, the more significant the mediating effect. The results reveal the “ability-cognition-behavior” driving mechanism behind older adults’ health information protection behavior, providing an integrated framework and intervention strategies for understanding the privacy protection behaviors of the older adult group in the digital age.

## Data Availability

The original contributions presented in the study are included in the article/supplementary material, further inquiries can be directed to the corresponding author.
